# NF-κB-mediated anti-inflammatory effects of an organic light-emitting diode (OLED) device in lipopolysaccharide (LPS)-induced *in vitro* and *in vivo* inflammation models

**DOI:** 10.3389/fimmu.2022.1050908

**Published:** 2022-12-06

**Authors:** SangJoon Mo, Eun Young Kim, Yi-Suk Kwon, Min Young Lee, Jin Chul Ahn

**Affiliations:** ^1^ Medical Laser Research Center, Dankook University, Cheonan, South Korea; ^2^ Center for Bio-Medical Engineering Core Facility, Dankook University, Cheonan, South Korea; ^3^ Beckman Laser Institute Korea, Dankook University, Cheonan, South Korea; ^4^ Korea Testing Laboratory, Medical Device Evaluation Center, Medical Health Division, Seoul, South Korea; ^5^ Department of Otolaryngology-Head & Neck Surgery, College of Medicine, Dankook University, Cheonan, South Korea

**Keywords:** inflammation, lipopolysaccharide, organic light emitting diode, photobiomodulation, nuclear factor kappa B

## Abstract

Inflammation is the body’s physiological response to harmful agents. However, if not regulated properly, inflammation can become pathological. Macrophages are key players in the inflammatory process, and modulate the immune response. Due to the side effects of anti-inflammatory drugs, non-pharmaceutical therapies for inflammatory diseases must be developed. Photobiomodulation is a non-invasive therapeutic approach to treating certain pathological conditions using light energy. Light-emitting diodes (LEDs) are commonly used as light sources for photobiomodulation treatment, but their clinical applications are limited. Organic LEDs (OLEDs) are thin, lightweight and flexible, enabling consistent and even delivery of light energy to target areas; this makes OLED promising components for therapeutic devices. In the present study, we examined the effects of OLED treatment on inflammation *in vitro* using a lipopolysaccharide (LPS)-induced macrophage RAW264.7 cell model, and *in vivo* using a pinna skin mouse model. We found that LPS-induced morphological changes and inflammatory cytokine expression were significantly reduced in RAW264.7 cells subjected to OLED treatment compared to the LPS-induced controls. This work provides evidence for the anti-inflammatory effects of OLEDs, demonstrating their potential to be incorporated into medical devices in the future.

## Introduction

Inflammation is an important immunological system involved in various physiological and pathological processes that is triggered by external stimuli, such as infection and tissue injury (1). Inflammatory responses can be acute, chronic, localized or systemic, and their pathomechanisms have been studied extensively (1). Macrophages are key components of the immune system, playing roles in antigen presentation and phagocytosis. In addition, they modulate immune responses during inflammation (2). When primary host defense systems such as the skin are breached, the inflammatory response is initiated, resulting in the recruitment of neutrophils and plasma to the site of infection or damage (2). In response to potential threats, nearby macrophages and mast cells are recruited to produce cytokines and chemokines, which can drive further inflammatory responses (2).

When regulated properly, inflammatory responses and processes benefit the human body by facilitating recovery from certain illnesses that would otherwise lead to severe morbidity or mortality. However, dysregulated inflammatory responses can cause various pathological conditions, such as septic shock and autoimmune responses (1). Therefore, regulating the inflammatory response is crucial for maintaining homeostasis. Inflammatory conditions are typically treated with pharmacological anti-inflammatory agents (3–6). However, these pharmaceuticals can cause serious complications, especially when used long term (7). Currently, novel treatments with fewer complications are being developed (4), including non-pharmacological therapeutics.

Photobiomodulation is a non-invasive therapeutic approach that uses light energy to treat certain pathological conditions (8) without altering normal physiology. Light energy can stimulate the proliferation and differentiation of certain cell types, and can thus be applied as a targeted treatment (9–11). Photobiomodulation has been demonstrated to confer anti-inflammatory effects in multiple organs, (12–14) and to trigger nuclear factor kappa B (NF-κB)−mediated responses (15).

Light-emitting diodes (LEDs) are commonly used as light energy sources to induce biological photomodulation (8). The clinical applications of LEDs for photobiomodulation are limited by a lack of LED delivery methods and difficulties in penetrating target tissue. Compared to conventional LEDs, organic LEDs (OLEDs) are thin, lightweight and flexible, enabling more effective and even delivery of light to target areas (16). Flexibility is an important characteristic of medical devices for the delivery of therapeutics to complex biological structures, such as the intestines and blood vessels, and for even distribution of therapeutics to curved areas such as the skin. Recently, Lee et al. reported wearable phototherapy skin patches that can suppress melanin production using microLEDs with 630 nm wavelength, but there are limitations in application and portability of various body parts because form factors are not more free than OLEDs (17). Thus, therapeutic photobiomodulation devices using OLEDs as a light source are a promising alternative light source for medical devices. A novel therapeutic skin patch employing OLEDs for photobiomodulation has recently been developed (18). Furthermore, OLEDs differ from standard LEDs in their physical properties, such as power and wavelength spectra; as a result, they may also differ in their therapeutic efficacy and mechanisms of action. Therefore, the effects and mechanisms of OLED treatments on inflammatory tissues and organs must be elucidated prior to their clinical application.

In this study, we investigated the anti-inflammatory effects of OLED treatment in an *in vitro* lipopolysaccharide (LPS)-induced macrophage RAW264.7 cell model, and in an *in vivo* ear pinna skin mouse model.

## Materials and methods

### Cell culture

Murine RAW264.7 macrophages were purchased from the American Type Culture Collection (ATCC, Manassas, VA, USA) and cultured in Dulbecco’s modified Eagle’s medium (DMEM; Corning, Tewksbury, MA, USA) containing 10% heat-inactivated fetal bovine serum (FBS; Corning) and 1% penicillin and streptomycin (Corning). Cells were incubated at 37°C in a humidified atmosphere with 5% CO_2_. LPS (List Labs, Campbell, CA, USA) was dissolved in distilled water to a stock concentration of 1 mg/mL and further diluted to 100 ng/mL in cell culture media.

### OLED irradiation

An OLED (WonTech, Daejeon, South Korea) with a wavelength of 630 nm was used to irradiate the LPS + OLED treatment group. OLED power was measured from the bottom of an empty plate prior to irradiation of cells with a VEGA ROHS power/energy meter (Ophir, Jerusalem, Israel) with a PD300-TP-ROHS detector head (Ophir). The OLED was directly irradiated outside the incubator for 10 minutes with an intensity of 4.5 mW/cm^2^ on the cell plate (total energy density: 2.7 J/cm^2^). Cell plates for control were also taken out of the incubator for the same period of time to equalize the conditions. For the mouse pinna inflammation model, OLED irradiation was conducted on 2 consecutive days at an intensity of 4.5 mW/cm^2^ for 10 min (total energy density: 5.4 J/cm^2^). Representative images of *in vitro* and *in vivo* applications of organic light emitting diode (OLED) devices and the parameters of the OLED light source are shown in **Supplementary Figure 1**.

### Epifluorescence imaging

RAW264.7 cells (3 × 10^5^ cells/well) were seeded onto cover slips in 6-well plates. After OLED irradiation, the cells on the cover slips were fixed in cold methanol for 10 min, and then permeabilized with 0.1% Triton X-100. The F-actin cytoskeleton was stained with phalloidin (Invitrogen, St. Louis, MO, USA) at a ratio of 1:40 for 30 min in the dark. Samples were then washed with phosphate-buffered saline (PBS), mounted on glass slides with VECTASHIELD mounting medium (Vector Laboratories, Burlingame, CA, USA), stained with DAPI and imaged using a confocal microscope (Olympus, Tokyo, Japan).

### qRT-PCR

Total RNA was extracted using TRIzol (GIBCO-BRL, Rockville, MD, USA). RNA concentrations were measured using a NanoDrop spectrophotometer (ND-1000; Nano Drop, Wilmington, DE, USA); 1 µg of total RNA was reverse-transcribed using oligo-dT primers and AccuPower^®^ RocketScript™ RT PreMix (Bioneer, Daejeon, Republic of Korea). qRT-PCR was performed using AccuPower^®^ 2× GreenStar™ qPCR Master Mix (Bioneer) in an RT-PCR system (ABI 7500; Applied Biosystems, Foster City, CA, USA). Relative mRNA expression levels were calculated using the formula: ΔCt = Ct (gene) - Ct. The 2^-ΔΔCt^ method was applied to calculate the fold-change of gene expression, which was normalized to GAPDH expression. The following primer pairs were used: GAPDH (forward: 5’-CCATCACCATCTTCCAGGAGCG-3’ and reverse: 5’-AGAGATGATGACCCTTTTGGC-3’), IL-1β (forward: 5’-TACAAGGAGAACCAAGCAACGACA-3’ and reverse: 5’-GATCCACACTCTCCAGCTGCA-3’), IL-6 (forward: 5’-CTTCCATCCAGTTGCCTTCTT-3’ and reverse: 5’-ACGATTTCCCAGAGAACATGT-3’), TNF-α (forward: 5’-ACGGCATGGATCTCAAAGAC-3’ and reverse: 5’-AGATAGCAAATCGGCTGACG-3’), iNOS (forward: 5’-AGTGGTGTTCTTTGCTTC-3’ and reverse: 5’-GCTTGCCTTATACTGGTC-3’), COX-2 (forward: 5’-GGTCTGGTGCCTGGTCTG-3’ and reverse: 5’-CTCTCCTATGAGTATGAGTCTGC-3’).

### Western blot

Total proteins were extracted using RIPA lysis buffer (50 mM Tris, pH 7.5, 150 mM NaCl, 0.5% sodium-deoxycholic acid, 0.1% sodium dodecyl sulfate, 1% Triton X-100 and 2 mM EDTA) containing 1% protease inhibitor and phosphatase inhibitors (Sigma-Aldrich, St. Louis, MO, USA). Protein concentrations were calculated using a DC protein assay kit (Bio-Rad, Hercules, CA, USA). Equivalent amounts of protein were subjected to electrophoresis on a 10% sodium dodecyl sulfate-polyacrylamide gel, and the separated proteins were electrotransferred onto polyvinylidene fluoride membranes (Bio-Rad). The membranes were blocked with blocking buffer containing 5% bovine serum albumin (BSA; Bioshop, South Korea) for 1 h and then incubated at 4°C overnight with primary antibodies (COX-2, iNOS, and β-actin; Cell Signaling Technology, Danvers, MA, USA). Membranes were washed with Tris-buffered saline Tween-20 (TBST; 20 mM Tris, pH 7.4, 150 mM NaCl and 0.1% Tween-20) and then incubated for 1 h at room temperature with secondary antibodies diluted in TBST (horseradish peroxidase [HRP]-anti-rabbit and HRP-anti-mouse; AB FRONTIER, Seoul, South Korea). The membranes were washed and then developed with a Clarity Western ECL substrate kit (Bio-Rad), and images were captured using a ChemiDoc XRS+ Imager (Bio-Rad).

### Immunofluorescence assay

RAW264.7 cells (3 × 10^5^ cells/well) were seeded in 6-well plates with cover slips overnight. After OLED irradiation, cells on the cover slips were fixed in cold methanol for 10 min, then permeabilized with 0.1% Triton X-100. After blocking in 5% BSA for 1 h, cells were incubated with primary antibodies against NF-κB p65 (Cell Signaling Technology) at 4°C overnight. The primary antibodies were removed by washing three times with PBS, and the samples were further incubated with secondary antibody (Alexa Fluor 488-conjugated goat anti rabbit IgG; Life Technologies, Carlsbad, CA, USA) for 1 h at room temperature. Finally, the cells were mounted with Vectashield mounting medium, stained with DAPI (Vector Laboratories) for visualization of the nucleus, and photographed under a confocal microscope (Olympus).

### Experimental design of mouse pinna inflammation model

Nine-week-old C57BL/6 mice (Nara Biotech, Inc., Seoul, South Korea) were used for the *in vivo* experiment. The breeding environment was a temperature of 23 ± 3°C, aa relative humidity of 50 ± 10%, a ventilation frequency of 10-20 times/h, and the light-dark cycle was adjusted in units of 12 h (illuminance 150-300 Lux). All animals were bred with solid feed (Purina: Nestle Purina PetCare Korea Ltd., Seoul, Seoul Korea) and water ad libitum, and all breeding equipment was sterilized. All animal experiments complied with the National Institutes of Health (NIH) regulations, and the experimental procedures were performed with the approval of the Animal Research Institute of Dankook University (IACUC) (DKU-20-008). Animals were divided into three groups: control group (n = 5), LPS-only group (n = 5), and LPS + OLED group (n = 5). First, 10 μL of LPS at a concentration of 1 mg/mL was injected into the LPS-only and LPS + OLED groups for 2 consecutive days; the same amount of sterile distilled water was injected into the control group. Commencing on day 2 of the LPS injection, the LPS + OLED group was irradiated with OLEDs for 2 consecutive days. Doppler scans (PeriScan PIM 3 System; PERIMED, Stockholm, Sweden) were performed after the final OLED irradiation. Pinna biopsies were fixed in 10% formalin overnight and embedded in paraffin. The tissues were stained with hematoxylin and eosin and toluidine blue. Immunohistochemistry was then conducted using Avidin-Biotinylated-Horseradish Peroxidase kits (Vector Laboratories) and a DAB-Detection System (Vector Laboratories). After deparaffinization, sections were treated and anti-neutrophil primary antibody, anti-CD11b antibody, anti-IL-1β antibody, anti-IL-6 antibody, and anti-TNF-α antibody (**Table 1**) were each diluted in 5% BSA and incubated at 4°C for 18 h. PBS was removed, and the appropriate biotinylated secondary antibody was added in 5% BSA (**Table 2**). Secondary antibody was incubated in sections for 1 h at room temperature. Secondary antibody was washed in PBS under gentle agitation for 5 min.

**Table 1:** Primary antibodies tested in mouse pinna tissues.

Target antigen	Supplier	Catalogue No.	Antibody raised in	Dilutions
**Neutrophil**	Abcam, UK	Ab2557	Rat	1/500
**CD11b**	MyBioSource, USA	MBS555372	Chicken	1/500
**IL-1β**	Abcam, UK	Ab205924	Rabbit	1/50
**IL-6**	Abcam, UK	Ab208113	Rabbit	1/200
**TNF-α**	GeneTex, USA	GTX110520	Mouse	1/100

**Table 2:** Secondary antibody reagents for mouse pinna tissues.

Target species	Supplier	Catalogue No.	Antibody raised in	Dilutions
**Rat**	Vector Laboratories, USA	BA-4000	Rabbit	1/500
**Chicken**	Vector Laboratories, USA	BA-9010	Goat	1/500
**Rabbit**	Vector Laboratories, USA	BA-1100	Horse	1/500

### Statistical analysis

All statistical analyses were performed with GraphPad Prism software (GraphPad Software Inc., San Diego, CA, USA) and SPSS software (IBM SPSS Statistics, New York). All data are presented as the mean ± standard deviation. Shapiro-Wilks normality tests with statistic specialist consultation (Kyung-Hwa Choi) (19, 20) were performed to determine whether the data were parametric or nonparametric. Levene’s tests were performed for equality of variance. One−way ANOVA or Kruskal-Wallis test (non-parametric distribution) with a *post hoc* Scheffe test (applied to both equal and unequal sample size comparisons) or Games-Howell test (unequal variance) or Dunn’s multiple comparison test (non-parametric distribution) were used for comparisons between groups (21). All statistics were reviewed by an institutional statistic specialist. P values less than 0.05 were considered to represent statistical significance. In the Figures, P values are shown as ^*^ *p* < 0.05, ^**^ *p* < 0.01 and ^***^ *p* < 0.001.

## Results

### OLED treatment decreased LPS-induced morphologic changes in RAW264.7 cells *in vitro*

**Figure 1:**
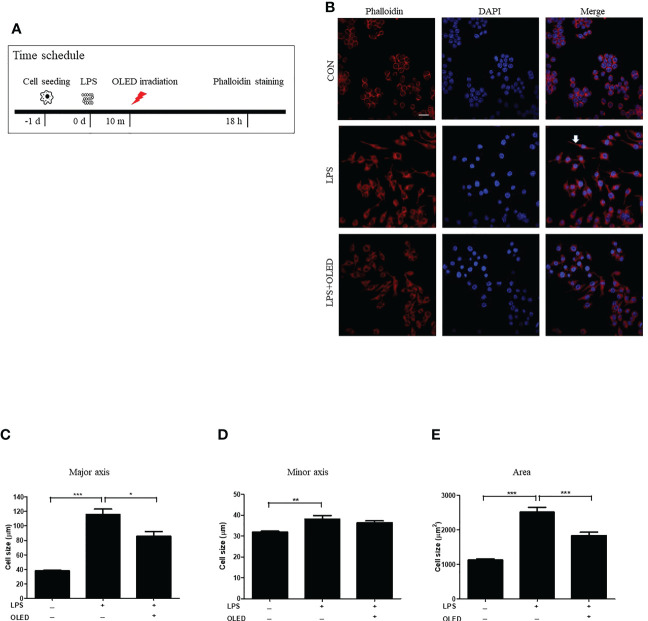
Epifluorescence analysis of lipopolysaccharide (LPS)-induced RAW264.7 cells after organic light-emitting diode (OLED) irradiation. **(A)** Experimental timeline for the epifluorescence analysis involving phalloidin staining after OLED irradiation in cells stimulated with LPS. **(B)** Images showing harvested cells stained with phalloidin (red) and 4′,6-diamidino-2-phenylindole (DAPI) (blue) for the visualization of actin fibers and nuclei. Following LPS application, cytoplasmic extensions (arrow) appeared. The number of cells exhibiting cytoplasmic extensions was lower with LPS and OLED treatment compared to treatment with LPS alone (scale bar: 20 μm). The major axial length of actin filaments was significantly lower in the OLED-treated compared to LPS-treated cells **(C)**. However, there was no significant difference in minor axial length **(D)** between the LPS-only and LPS+OLED treatment groups. **(E)** The cell area was significantly smaller in OLED-treated compared to LPS treated cells. Control (n = 84), LPS (n = 33), LPS + OLED (n = 48); ^*^ *p* < 0.05, **p < 0.01 (control group vs. LPS-only group); ^***^ *p* < 0.001 (LPS+OLED group vs. LPS-only group).

LPS exposure causes RAW264.7 cells to undergo morphological changes (22, 23), including the cytoplasmic extensions and an increase in cellular size. Here, we compared the morphological and inflammatory responses of RAW264.7 cells without LPS treatment (control group), with LPS stimulation only (LPS-only group) and with LPS stimulation and subsequent OLED treatment (LPS + OLED group). RAW264.7 cells were cultivated for 24 h and then treated with LPS; cells in the LPS + OLED group were irradiated for 10 min after LPS treatment. Cells were harvested 18 h after LPS treatment (**Figure 1A**) and stained with phalloidin and 4′,6-diamidino-2-phenylindole (DAPI) for fluorescence imaging. Cytoplastic extensions appeared on the cells after LPS treatment. However, the number of cells exhibiting cytoplasmic extensions was lower in the LPS + OLED group compared to the LPS-only group (**Figure 1B**).

The length of each cell was measured in the major and minor axes, and cell areas were calculated using Image J software (11). The major cellular axis length was significantly larger in the LPS-only group compared to the LPS + OLED and control groups (one-way analysis of variance ([ANOVA with Welch test], *p* < 0.0001; F = 81.62; df = 159; control (n = 81), LPS (n = 33), LPS + OLED (n = 48). Games-Howell test: control vs. LPS-only, *p* < 0.001; LPS-only vs. LPS + OLED, *p* = 0.011) (**Figure 1C**); the minor axis was significantly longer in the LPS-only group compared to the control group (one-way ANOVA, *p* < 0.0001; F = 14.45; df = 161; control (n = 84), LPS (n = 30), LPS + OLED (n = 48). Scheffe test multiple comparison test: control vs. LPS-only, *p* = 0.003; LPS-only vs. LPS + OLED, *p* = ns) (**Figure 1D**). Furthermore, cellular area was significantly larger in the LPS-only group compared to the control and LPS + OLED groups (one-way ANOVA with Welch test, *p* < 0.0001; F = 89.77; df = 162; control (n = 83), LPS (n = 32), LPS + OLED (n = 47). Games-Howell test: control vs. LPS-only, *p* < 0.001; LPS-only vs. LPS + OLED, *p* < 0.001) (**Figure 1E**). These results indicate that morphological changes occurred in the RAW264.7 cells as a result of LPS application, and that OLED reduced the LPS-induced morphological changes.

### OLED treatment reduced the expression of LPS-induced inflammatory mediators in RAW264.7 cells

**Figure 2:**
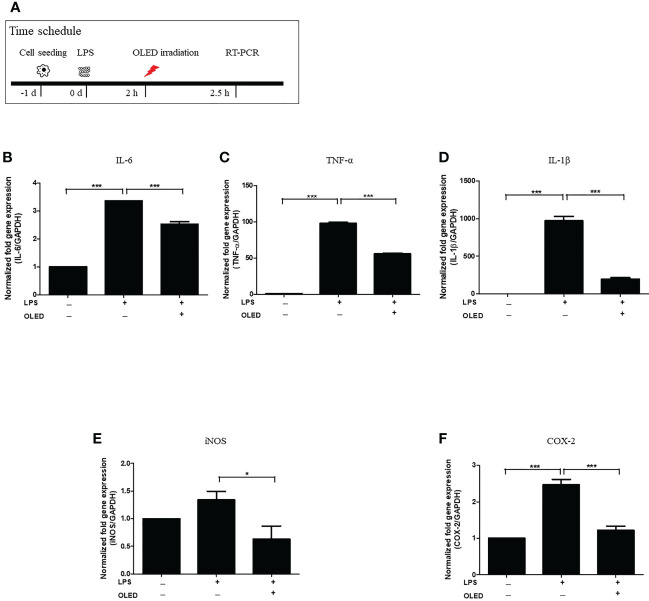
mRNA levels of inflammatory mediators in lipopolysaccharide (LPS)-induced RAW264.7 cells after organic light-emitting diode (OLED) irradiation. **(A)** Experimental timeline for examination of the relative mRNA expression levels of interleukin (IL)-6, tumor necrosis factor-α (TNF-α), IL-1 β, inducible nitric oxide synthase (iNOS) and cyclooxygenase-2 (COX-2) revealed by quantitative real-time PCR after OLED irradiation of LPS-stimulated cells. The relative mRNA levels of IL-6 **(B)**, TNF-α **(C)**, IL-1β **(D)**, iNOS **(E)** and COX-2 **(F)** are shown. OLED irradiation significantly reduced the mRNA levels of inflammatory mediators. n = 3; ^*^ *p* < 0.05, ^***^ *p* < 0.001 (LPS+OLED group vs. LPS-only group).

To examine the expression levels of macrophage-specific pro-inflammatory mediators and cytokines following LPS and OLED treatment, we performed quantitative real-time polymerase chain reaction (qRT-PCR) on the control, LPS-only and LPS + OLED groups 2.5 h after LPS treatment. Cells were subjected to OLED treatment 2 h after LPS application in the LPS + OLED group (**Figure 2A**). The mRNA levels of the inflammatory mediators interleukin (IL)-6, tumor necrosis factor-α (TNF-α), IL-1 β, inducible nitric oxide synthase (iNOS) and cyclooxygenase-2 (COX-2) are shown for each group in **Figures 2B–F**. The levels of four mRNAs (IL-6, TNF-α, IL-1 β and COX-2) were significantly higher in the LPS-only group compared to the control group (one-way ANOVA: IL-6, *p* = 0.0001, F = 498.4, df = 8, n = 3; TNF-α, *p* = 0.0001, F = 1632, df = 8, n = 3; IL-1β, *p* = 0.0001, F = 228.1, df = 8, n = 3; COX-2, *p* = 0.0001, F = 53.72, df = 8, n = 3. Scheffe test: IL-6, *p* < 0.0001; TNF-α, *p* < 0.0001; IL-1β, *p* < 0.0001; COX-2, *p* = 0.0002). The levels of all mRNAs examined (IL-6, TNF-a, IL-1 β, iNOS and COX-2) were significantly lower in the LPS + OLED group compared to the LPS-only group [one-way ANOVA: IL-6, *p* = 0.0001, F = 498.4, df = 8, n = 3; TNF-α, *p* = 0.0001, F = 1632, df = 8, n = 3; IL-1β, *p* = 0.0001, F = 228.1, df = 8, n = 3; iNOS1, *p* = 0.0251, F = 4.756, df = 17, n = 6; COX-2, *p* = 0.0001, F = 53.72, df = 8, n = 3. Scheffe test: IL-6, *p* < 0.0001; TNF-α, *p* < 0.0001; IL-1β, *p* < 0.0001; iNOS, *p* = 0.025; COX-2, *p* = 0.001).

**Figure 3:**
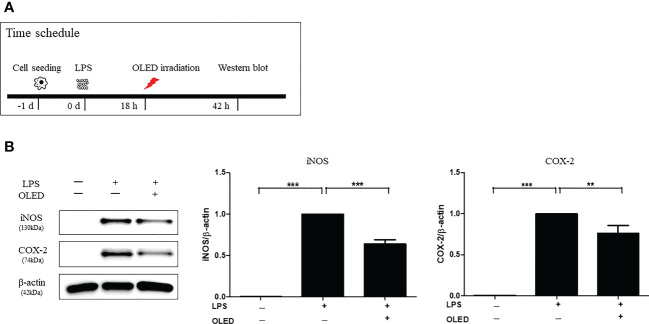
Western blots showing the protein expression levels of inducible nitric oxide synthase (iNOS) and cyclooxygenase-2 (COX-2) in lipopolysaccharide (LPS)-induced RAW264.7 cells after organic light-emitting diode (OLED) irradiation. **(A)** Experimental timeline for the Western blot analysis examining the expression levels of iNOS and COX-2 proteins following OLED irradiation. **(B)** The protein levels of iNOS and COX-2 were significantly lower in OLED-treated compared to LPS-treated cells. n = 5; ^**^p < 0.01, ^***^p < 0.001 (LPS+OLED group vs. LPS-only group).

Next, we performed Western blots for each experimental treatment group of RAW264.7 cells 42 h after LPS treatment to examine iNOS and COX-2 protein expression. OLED treatment was applied 18 h after LPS treatment in the LPS + OLED group (**Figure 3A** and **Supplemental Figure 2**). The iNOS and COX-2 protein expression levels for the control, LPS-only and LPS + OLED groups are shown in **Figure 3B**. The protein expression levels of iNOS and COX-2 were significantly higher in the LPS-only group compared to the control group (one-way ANOVA: iNOS, *p* = 0.0001, F = 283.9, df = 14, n = 3; COX-2, *p* < 0.0001, F = 91.04, df = 14, n = 3. Scheffe test: iNOS, *p* < 0.0001; COX-2, *p* < 0.0001) and compared to the LPS + OLED group (Scheffe test: iNOS, *p* < 0.0001; COX-2, *p* = 0.003). Taken together, these results suggest that the inflammatory responses and morphological changes that take place after LPS treatment are mitigated by OLED therapy.

### LPS-induced nuclear translocation of NF-κB is reduced in RAW264.7 cells following OLED treatment

**Figure 4:**
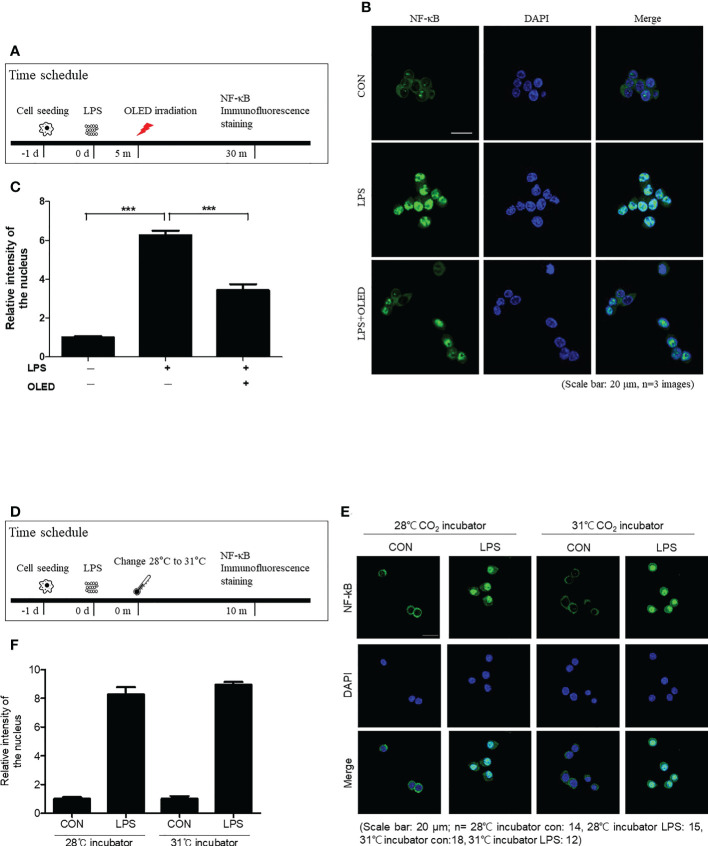
The nuclear translocation of nuclear factor kappa B (NF-κB; p65) after organic light-emitting diode (OLED) irradiation in the lipopolysaccharide (LPS)-stimulated RAW264.7 cells. **(A)** Experimental timeline for the analysis of NF-κB (p65) localization by immunofluorescence staining. **(B)** Immunofluorescence images showing p65 localization (green) and 4′,6-diamidino-2-phenylindole (DAPI)-stained nuclei (blue) in LPS-induced RAW264.7 cells. In the control cells (no LPS stimulation), basal p65 was distributed throughout the cytoplasm. Following LPS stimulation, nuclear translocation of p65 can be observed (merged blue and green colors). OLED irradiation disrupted the nuclear translocation of p65 in LPS-induced RAW264.7 cells. Scale bar: 20 μm. **(C)** Quantification of the relative fluorescence intensities of p65 localized to nuclei. The LPS-only group exhibited significantly higher p65 nuclear fluorescence compared to LPS+OLED. n = 3; ^***^ *p* < 0.001 (LPS+OLED group vs. LPS-only group). **(D)** The experimental schedule for comparing NF-κB expression pattern (30 min after LPS) between two different culture conditions (28°C and 31°C for 10 min) is shown. **(E)** LPS application induced nuclear translocation of NF-κB in both temperature conditions. **(F)** No statistical difference between temperatures in both control (without LPS) and LPS groups was observed.

The transcription factor NF-κB regulates the expression of several genes involved in the inflammatory response (24, 25). To investigate the mechanisms driving the anti-inflammatory effects of OLED treatment in LPS-induced RAW264.7 cells, we performed epifluorescence microscopy to examine NF-κB expression and localization in the control, LPS-only and LPS + OLED groups. OLED treatment was performed 5 min after LPS application in the LPS + OLED group. Epifluorescence analysis was performed 30 min after LPS application (**Figure 4A**). In the control group, NF-κB (green) was clearly present in the cytoplasm. By contrast, NF-κB (green) localized to the DAPI-stained nuclei (blue) in the LPS-only group, which was in accordance with previous reports of NF-κB nuclear translocation upon LPS stimulation in RAW264.7 cells (26). For the LPS + OLED group, NF-κB was present in both the cytoplasm and nucleus (**Figure 4B**). We then compared the relative intensities of nuclear NF-κB among the experimental groups; significantly higher NF-κB fluorescence intensities were observed in the nuclei of the LPS-only group compared to the nuclei of the control and LPS + OLED groups (Kruskal-Wallis test: *p* = < 0.0001, KW statistic = 65.2, n = 26−32. Dunn’s multiple comparison test: control vs. LPS-only, *p* < 0.0001; LPS-only vs. LPS + OLED, *p* = 0.0005) (**Figure 4C**). These results suggest that OLED treatment disrupts the nuclear translocation of NF-κB in LPS-induced RAW264.7 cells. To confirm these outcomes further, protein ratio of p- NF-κB/NF-κB and p-AKT/AKT (which is expressed earlier than NF-κB) were accessed by Western blot analysis. Statistically significant difference was not observed in NF-κB protein expressions. While significant difference was observed in AKT expression ratio, statistically higher ratio of p-AKT/AKT were observed in LPS group compared to control and OLED group (one-way ANOVA: *p* = 0.0013, F = 12.24, Scheffe test: control vs. LPS-only, *p* = 0.002; LPS-only vs. LPS + OLED, *p* = 0.002) (**Supplemental Figure 3** and **Figure 4**). Due to the relative large temperature deviation (**Figure S1**) by OLED application which might trigger cell response and alter cytokine production, we have included the comparison NF-κB expression pattern (30 min after LPS) between two different culture condition which differs only temperature (28°C and 31°C for 10 min) (**Figure 4D**). In both temperature conditions, LPS application induced nuclear translocation of NF-κB (**Figure 4E**). Reduced nuclear translocation which was observed in the OLED + LPS group was not observed. There was no statistical difference between temperatures in both control (without LPS) and LPS groups (**Figure 4F**).

### OLED treatment dampened the inflammatory response after LPS injection in mouse pinna

**Figure 5:**
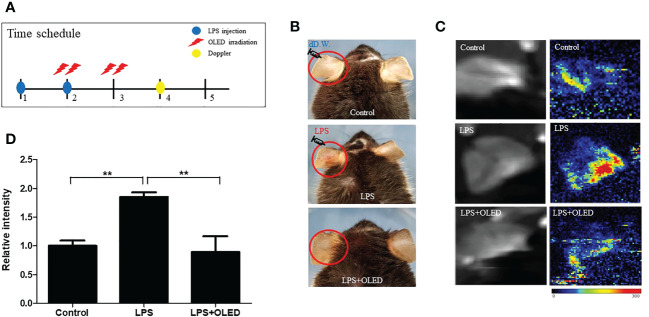
The effect of organic light-emitting diode (OLED) irradiation on blood flow in lipopolysaccharide (LPS)-injected mouse ear pinnae. **(A)** Experimental timeline for the experiments. **(B)** Representative photographs of ear pinnae in C57BL/6 mice injected with LPS. The pinnae of LPS-only mice were more hyperemic than LPS+OLED mice. **(C)** Laser Doppler images of mouse ear pinnae. The pinnae of LPS+OLED mice exhibited reduced blood flow compared to the LPS-only group. **(D)** Quantitative analysis of tissue blood flow in Doppler images revealed significantly reduced blood flow in the control and LPS+OLED groups compared to the LPS-only group. DW, distilled water; n = 3; ^**^p < 0.01 (LPS+OLED group vs. LPS-only group).

**Figure 6:**
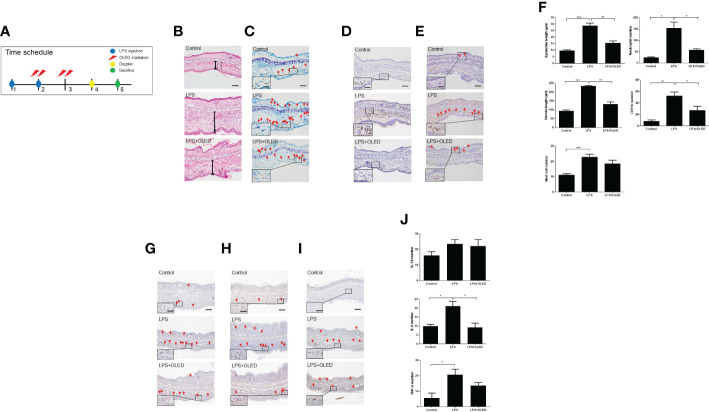
The effect of organic light-emitting diode (OLED) irradiation on the histological features of lipopolysaccharide (LPS)-injected mouse ear pinna tissue. **(A)** Timeline for the experiments. **(B)** Images of ear pinna tissues stained with hematoxylin and eosin. Numbers 1 and 2 indicate the epidermis and dermis, respectively. The pinna epidermal layers are thicker in the LPS-only group compared to the control and LPS+OLED groups. **(C)** Representative images of ear pinnae collected from each group stained with toluidine blue. Arrows indicate mast cells. The number of mast cells was lower in the control tissue compared to the LPS-only and LPS+OLED groups. Moreover, the number of mast cells was lower in the LPS+OLED pinna tissue compared to the LPS-only group; however, the difference was not significant. **(D)** Images of ear tissue sections immunostained for neutrophils. The number of neutrophils was lower in LPS+OLED ear pinna tissue compared to the LPS-only group. **(E)** Images of ear tissue sections immunostained for CD11b. The number of CD11b was lower in LPS+OLED ear pinna tissue compared to the LPS-only group. Scale bar: 100 μm, magnified image scale bar: 5 μm. **(F)** The histological immunofluorescence staining was quantified and the data presented here are the mean ± standard error of mean (SEM). Compared to the LPS-only group, the epidermal and dermal layers were significantly thinner, the neutrophil and the macrophage counts were significantly lower in the pinna tissue of the LPS+OLED group. n = 5; ^*^ *p* < 0.05, ^**^ *p* < 0.01 vs. LPS-only group. ***p < 0.001 (n=5, control group vs. LPS-only group). Images of ear tissue sections immunostained for **(G)** IL-1β; **(H)** IL-6; **(I)** TNF-α. Scale bar: 100 μm. **(J)** Quantitation of the histological analysis levels were presented as the mean ± SEM. Quantification of these data showed differences between groups and was statistically lower in the OLED irradiation group compared to the LPS induced group. (control: n = 3, LPS, LPS+OLED: n=5; ^*^ *p* < 0.05, versus LPS injection group).

To investigate the anti-inflammatory effects of OLED *in vivo*, a mouse skin inflammation model was employed. Mice were divided into three groups: control, LPS-only and LPS + OLED. LPS (10 μL, 1 mg/mL) was injected into the ear pinnae of mice in the LPS-only and LPS + OLED groups on two consecutive days; the same amount of distilled water was injected into control group mice. Mice in the LPS + OLED group were subjected to OLED treatment on the second day of LPS injection for 2 consecutive days. Doppler examinations were performed 1 day after OLED treatment, and a histological evaluation of the immune response was performed 2 days after OLED treatment (**Figure 5** and **Figure 6A**). On experimental day 4, the pinnae of mice from the LPS-only group exhibited hyperemia (reddish color change), whereas the pinnae of control and LPS + OLED mice exhibited a normal skin color (**Figure 5B**). The doppler examination revealed higher vascular blood flow in the pinnae of LPS-only mice compared to those of LPS + OLED and control mice (**Figure 5C**). Relative intensities were measured and averaged (n = 3 for each experimental group); the mean intensity was significantly higher in the LPS-only group compared to the control and LPS + OLED groups (one-way ANOVA: *p* = 0.0149, F = 9.195, df = 8, n = 3. Scheffe test: control vs. LPS-only, *p* = 0.006; LPS-only vs. LPS + OLED, *p* = 0.005) (**Figure 5D**).

Next, we performed a histological examination of pinna tissue samples collected from mice in each experimental group to compare the inflammatory responses to LPS and OLED treatment. Dermal and epidermal thickness were measured (27–29) and the numbers of mast cells, leukocytes and macrophages were quantified (30, 31). Hematoxylin and eosin staining of pinna tissues revealed that both the dermis and epidermis were thicker in the LPS-only group compared to the control and LPS + OLED groups (**Figure 6B**). Toluidine-blue staining revealed that the number of mast cells was higher in pinna skin samples taken from LPS-only mice compared to control and LPS + OLED pinna samples (**Figure 6C**). Furthermore, the population of neutrophils and macrophages (stained for CD11b) was denser in the LPS-only pinna samples compared to those of the control and LPS + OLED groups (**Figures 6D, E**). Statistical analyses of the above findings revealed that the dermis and epidermis were significantly thicker, and the numbers of mast cells, neutrophils and macrophages were statistically higher, in the LPS-only group compared to the control (**Table 3** and **Figure 6F**). Moreover, the dermis and epidermis were significantly thinner, and the number of neutrophils and macrophages were significantly lower, in the pinnae of the LPS + OLED group compared to those of the LPS-only group (**Table 3** and **Figure 6F**). Moreover, immunostains confirmed that increased inflammatory cytokines by LPS injection (IL-6, TNF-a) was reduced (**Figures 6G–I**). IL-6 showed statistically significant reduction (**Figure 6J** and **Table 3**). The findings from these *in vivo* studies further support our *in vitro* data, which together suggest that OLED treatment can mitigate LPS-induced inflammation both *in vitro* and *in vivo*.

**Table 3:** Statistical analysis of histology after OLED irradiation.

	One way ANOVA (P value)	Control vs LPS	LPS vs LPS + OLED
Dermis thickness	< 0.0001*	< 0.0001*	0.002*
Epidermis thickness	< 0.0001*	0.001*	0.002*
Mast cells number	0.001**	0.001**	0.279
Neutrophils number	0.0002*	0.018*	0.049*
Macrophage number	0.002**	0.002**	0.040**
IL-6	0.007**	0.033**	0.011**
TNF-α	0.027**	0.023**	0.2403

*One way ANOVA with Welch test for unequal variance (post hoc Games-Howell test). **One way ANOVA (post hoc Scheffe test).

Bold: statistically significant.

## Discussion

**Figure 7:**
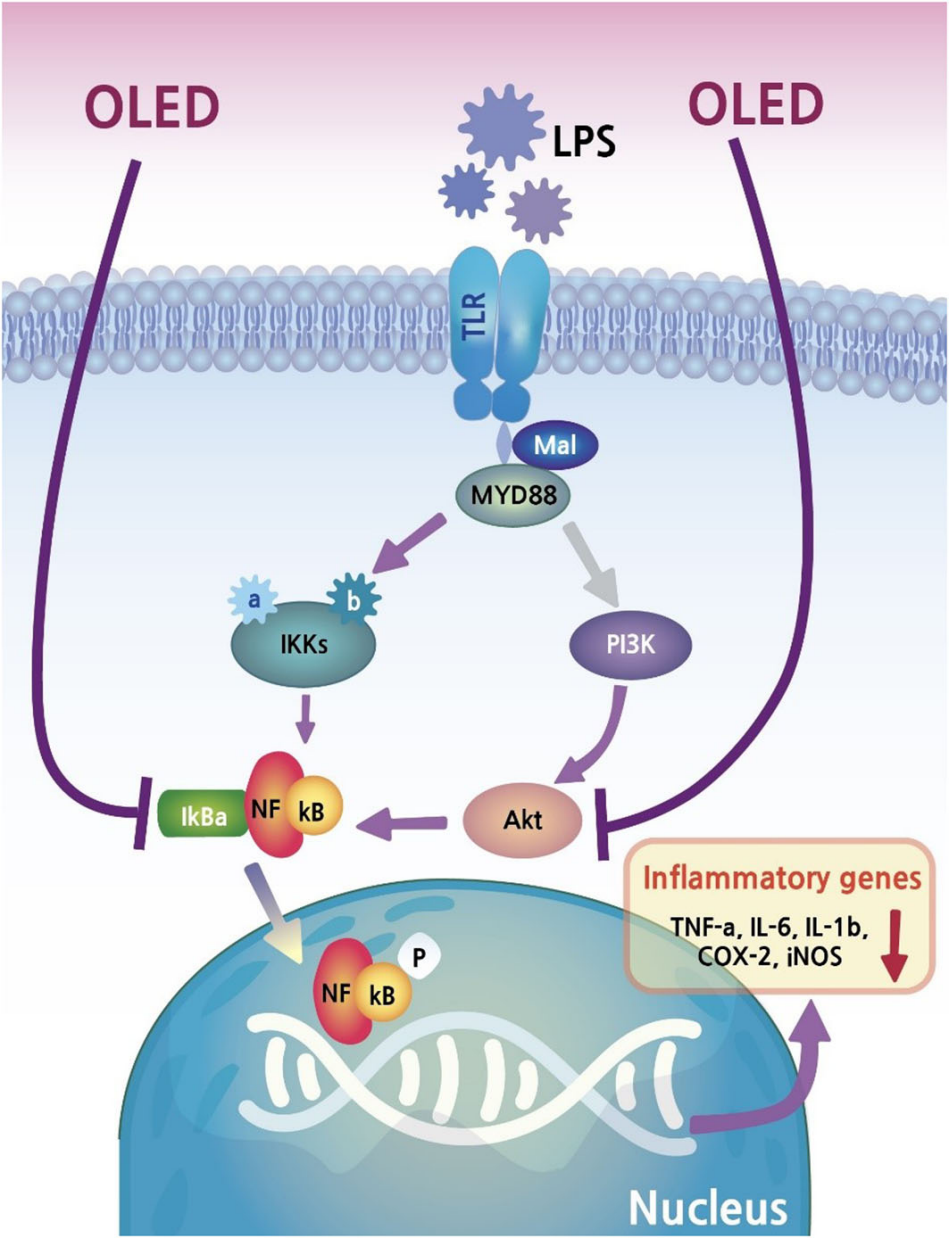
The potential inflammatory suppression mechanism of PBM.

In the present study, we found that LPS exposure in the inflammatory cell line RAW264.7 triggered morphological changes, and that these changes were inhibited by photobiomodulation (OLED) treatment. As discussed above, OLEDs possess properties such as flexibility, making them more appropriate for certain medical devices than conventional LEDs. Our *in vitro* study provided evidence that OLEDs can confer anti-inflammatory effects on a common inflammatory cell line. To investigate this in more detail, we quantitatively assessed the mRNA and protein levels of inflammatory mediators in OLED-treated cells; we found that LPS treatment alone caused upregulation of inflammatory cytokines in the macrophage cell line, and that OLED treatment significantly mitigated this effect. Moreover, the nuclear translocation of NF-κB, one of the key factors regulating inflammation in response to LPS, was inhibited in cells treated with OLED. Our results provide evidence for the anti-inflammatory effects of OLED photobiomodulation treatment, as well as insight into the possible mechanisms underlying this response (**Figure 7**).

We then investigated the effects of OLED treatment using an *in vivo* animal model, in which inflammation was induced in mouse ears by injecting pinnae with LPS; the physiological effects of subsequent OLED treatment were then examined. Following LPS injection, we observed inflammatory responses in mouse ear pinnae such as soft tissue thickening, increased blood flow and the recruitment of inflammatory cells. Notably, these responses were reversed or inhibited in mice subjected to OLED treatment. The difference between the *in vitro* and *in vivo* study exists (inflammatory cytokines). It would be due to experimental time points. Since *in vivo* studies were performed at a later stage, it is possible that acute phase cytokines could have been normalized. Taken together, the results from the *in vitro* and *in vivo* experiments suggest that OLED treatment reduces inflammation. It is not clear whether this positive effect is temporary delay or cessation of long term inflammation. According to our data we can speculate that this response would last longer than 4 days since the *in vivo* analysis was mostly performed at 3 to 4 days time points and shows reduction of inflammation signs. Considering the fact that acute inflammation responses are defined as reaction lasting for minutes to hours to days, it is highly likely that OLED PBM is not just delaying the process.

The clinical applications of OLED treatment include the treatment of conditions in which inflammation is pathological and cannot be regulated by the patients’ natural homeostatic systems. Currently, medications are used to treat such inflammatory diseases; however, anti-inflammatory drugs are typically non-specific and can cause severe side effects. OLED devices can potentially overcome the current limitations of conventional LED-based photobiomodulation devices, which include limited access to target tissues and low penetration rates. Flexible OLED devices could benefit patients experiencing uncontrolled inflammation in specific tissues that cannot currently be accessed by photobiomodulation delivery apparatuses.

After 10 min irradiation, which is the duration of OLED exposure in our experiment, about 2~3-degree increase was observed in the experimental model measuring temperature changes (**Figure S1**). However, there was a difference between actual experiment and temperature measurement. Since the OLED irradiation was performed outside the incubator, plastic cover was applied to minimize the contamination (plastic cover did not alter OLED intensity; **Figure S1H**). On the other hand, for the temperature measurement, plastic cover could not be applied. We strongly believe that this plastic cover would minimize the thermal conduction to cells in actual experiments. Extremely minimal change of temperature in areas ‘outside (number 2 and 3)’ which is closer to the OLED board supports the theory. In addition, no difference in nuclear translocation of NF-κB (**Figure 4E**) between the two temperatures supports the no thermal effect theory. It is rarely possible but even though this thermal change was made in actual experiment, temperature was below 35 degree which is the below thermal threshold for activation of heat shock proteins (32) and immune cell activation (33). These both responses will promote inflammation which is opposite to the outcome of current study (down regulation of inflammatory response). Several models have been proposed to explain the anti-inflammatory effects of photobiomodulation, including mitochondrial activation by the light-responsive cytochrome c oxidase. In this model, ATP production is increased following mitochondrial activation, which in turn may alter the inflammatory pathways regulated by NF-κB.

Lasers are currently the dominant light sources for photobiomodulation. Until recently, lasers were thought to exert biologically beneficial effects; however, recent studies have suggested otherwise (34, 35). The major difference between lasers and LEDs is the bandwidth. LED-based light therapy has recently been established in many healthcare centers due to the lower cost and larger possible treatment area of LEDs compared to lasers. Photobiomodulation using LEDs has been actively researched since 2001, and is now widely accepted to be an effective therapy (8). Flexible and wearable LED devices for photobiomodulation treatment could be developed using OLEDs, which were shown to be effective in this study.

Several studies have been conducted to investigate the wavelength-specific effects of lasers and conventional LEDs on various cell lines and organs. However, *in vitro* studies investigating OLED systems are limited. In a recent study, OLED treatment with 630−690 nm wavelength and 10−30 min exposure time caused an increase in cellular activity. In addition, OLEDs with 630−650 nm wavelengths were effective at lower powers (3−6 J/cm^2^), whereas those with 670−690 nm wavelengths were more effective at higher powers (6−9 J/cm^2^) (18). Therefore, the OLED parameters used in the present study (630 nm wavelength, 10 min exposure at 5 J/cm^2^ power) were selected to optimize the effects on cell lines and tissues.

Invasive pathogens such as bacteria, viruses and fungi trigger cell differentiation, and the production of cytokines and proteases in monocytes and macrophages (36). LPS is a macromolecule comprising lipids and polysaccharides that is present in the cell walls of Gram-negative bacteria (37). LPS is recognized *via* Toll-like receptors (TLRs), which stimulate a potent immune response (38). In particular, TLR2 and TLR4 act as key sensors for the immune system, triggering a counter response to the invasion of pathogenic bacteria (39). The binding of LPS to TLR2 and TLR4 activates the NF-κB signaling pathway, which induces the transcription of pro-inflammatory mediators such as IL-1β, IL-6, TNF-α, prostaglandin E2, iNOS and COX-2. iNOS and COX-2 are considered to be major inflammatory mediators; however, their overexpression can have detrimental effects on cells (40–42).

Treating RAW264.7 cells with LPS induces morphological changes, such as an increase in cell size. In addition, Guo et al. found that NF-κB drives LPS-induced morphological changes in RAW264.7 cells by regulating the actin cytoskeleton (43, 44). In the present study, we found that the LPS-induced morphological changes in RAW264.7 cells were partially suppressed by OLED treatment. This led us to hypothesize that OLED irradiation prevented cytoskeletal restructuring by inhibiting NF-κB signaling.

The anti-inflammatory effects of OLED treatment were further demonstrated *via* the inhibition of nitric oxide (NO) production, where NO is an important mediator of inflammatory reactions such as phagocytosis. A reduction of pro-inflammatory factors by LED irradiation has previously been reported; however, OLED-induced reductions in the expression of pro-inflammatory cytokines, such as COX-2 and iNOS, were reported in this study for the first time (45). Notably, we compared the anti-inflammatory effects of LED and OLED treatment, and found that OLED treatment inhibited the expression of some inflammatory factors to a greater extent than LED treatment (data not shown).

The immunomodulatory effects of OLED treatment were reflected in the suppression of NF-κB localization in OLED-treated cells. The NF-κB pathway plays an essential role in inflammation and responses to cellular damage (46). Upon induction of the inflammatory response by LPS, genes related to inflammation are upregulated following the translocation of NF-κB from the cytoplasm to the nucleus (46). However, in OLED-irradiated cells in this study, the process of NF-κB nuclear migration was inhibited; therefore, we inferred that the expression and secretion of pro-inflammatory cytokines was also suppressed. The findings from our *in vivo* animal experiments further supported this hypothesis. We found that mouse ear pinnae injected with LPS exhibited increased blood flow. and dermal and epidermal thickening. due to capillary expansion and immune cell recruitment; however, this response was suppressed in mice treated with OLED following LPS injection. Furthermore, tissue staining revealed a reduction in the number of mast cells and neutrophils in OLED-treated ear tissue compared to ears treated with LPS only. In another study, the present authors inhibited cell death by effectively inhibiting the generation of ROS under H_2_O_2_-induced oxidative stress by irradiating LEDs of the same wavelength (under revision). Based on this, when OLED is irradiated to the inflammation-induced tissue, it can be inferred that the inhibition of ROS production by neutrophil production ultimately suppresses the inflammatory response (47). It is strongly predicted that OLED irradiated after antioxidant treatment would inhibit the NF-kB expression pathway as well. In addition, there is a report showing a combination effect of antioxidant and PBM conditioning in auditory hair cells which shows partial synergistic effect (48). But for this current experiment, seeking the combination effect was not the purpose.

The anti-inflammatory effects of LEDs have been demonstrated for a variety of diseases; however, investigations into the effects of OLEDs have tended to focus on wound healing (18). In this study, we found that OLEDs can elicit similar anti-inflammatory responses to LEDs. OLEDs and conventional LEDs differ in their physical properties; as OLEDs have the ability to distribute light over curved surfaces with a uniform energy intensity and wide spectral bandwidth, they have many promising applications in the medical field (S Video 1). Therefore, with more following researches proving the efficiency of the OLED application to various inflammatory conditions, development of flexible medical devices using OLED for controlling the inflammatory responses in near future is expected.

## Data availability statement

The original contributions presented in the study are included in the article/**Supplementary Material**. Further inquiries can be directed to the corresponding authors.

## Ethics statement

The animal study was reviewed and approved by Animal Research Institute of Dankook University (IACUC) (DKU-20-008).

## Author contributions

SM and EYK performed the experiment. SM, MYL, and JCA analyzed the results. SM and JCA designed all the experiments. SM, EYK, and MYL wrote the manuscript. MYL and JCA edited the manuscript. Y-SK analyzed the photophysical properties of OLED. All Authors contributed to the revision of the manuscript and approved the final version for publication. All authors contributed to the article and approved the submitted version.
