# Supplementary material for: NF-κB-mediated anti-inflammatory effects of an organic light-emitting diode (OLED) device in lipopolysaccharide (LPS)-induced in vitro and in vivo inflammation models
Source: Front Immunol. 2022 Dec 6;13:1050908. doi: 10.3389/fimmu.2022.1050908 (PMC9763281; doi:10.3389/fimmu.2022.1050908)
Supplement: Supplementary file 1 [file DataSheet_1.docx]

**Supplementary material**

**NF-κB-mediated anti-inflammatory effects of an organic light-emitting diode (OLED) device in lipopolysaccharide (LPS)-induced *in vitro* and *in vivo* inflammation models**

**Supplementary figure 1. Representative images of the organic light-emitting diode (OLED) device *in vitro*, and *in vivo* applications and** **OLED light source parameters**

**
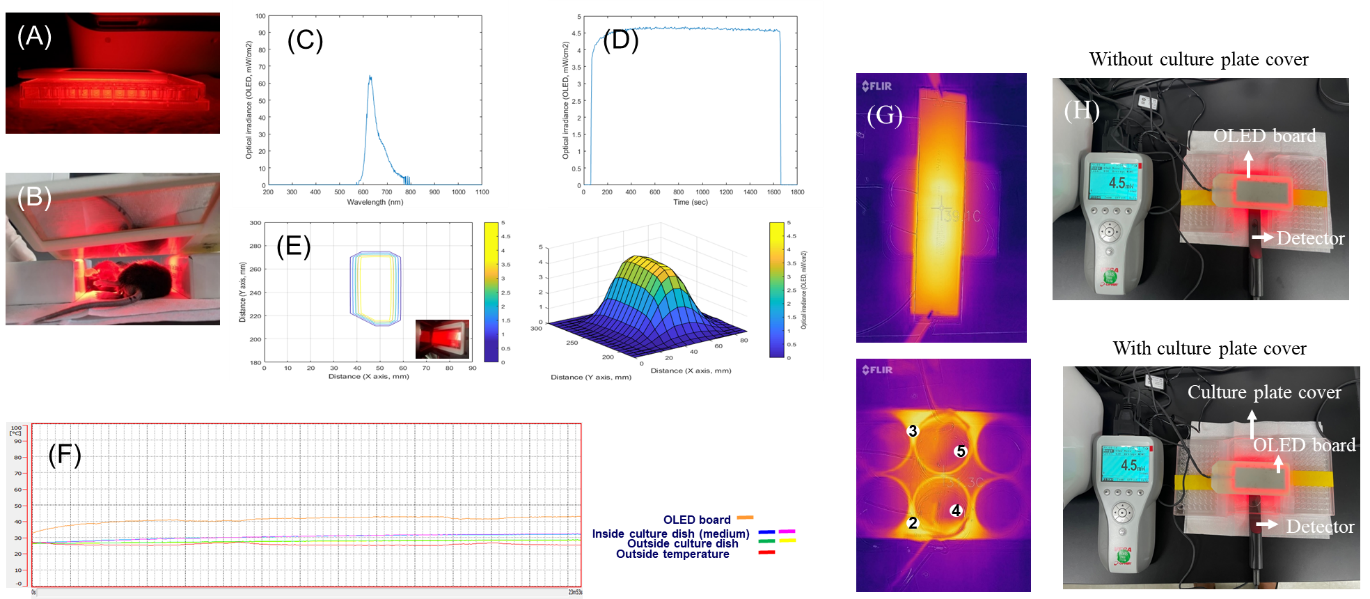
**

**Figure S1.** (A) RAW264.7 cells on a 96-well plate were exposed to red OLED light irradiation. (B) Red OLED light irradiation of mouse ear pinnae. (C) The optical irradiance and wavelength of the red OLED. (D) Graph showing the changes in optical irradiance of the red OLED over irradiance time. Optical irradiance was measured using a spectroradiometer. (E) Two- and three-dimensional plots showing the OLED beam characteristics. (F) Graph showing changes caused by OLED emission (Plastic cover not applied; applied in actual experiment). (G) During OLED irradiation, temperature measurement point. ①: OLED board, ② and ③: Outside the culture plate, ④ and ⑤: Inside the culture plate (culture medium). (H) Intensity of red OLED with and without a culture plate cover.

**Supplementary figure 2. Uncropped images of western blot analysis (inflammatory markers)**

**Supplementary figure 3. Western blot analysis of NF-κB and AKT**

**
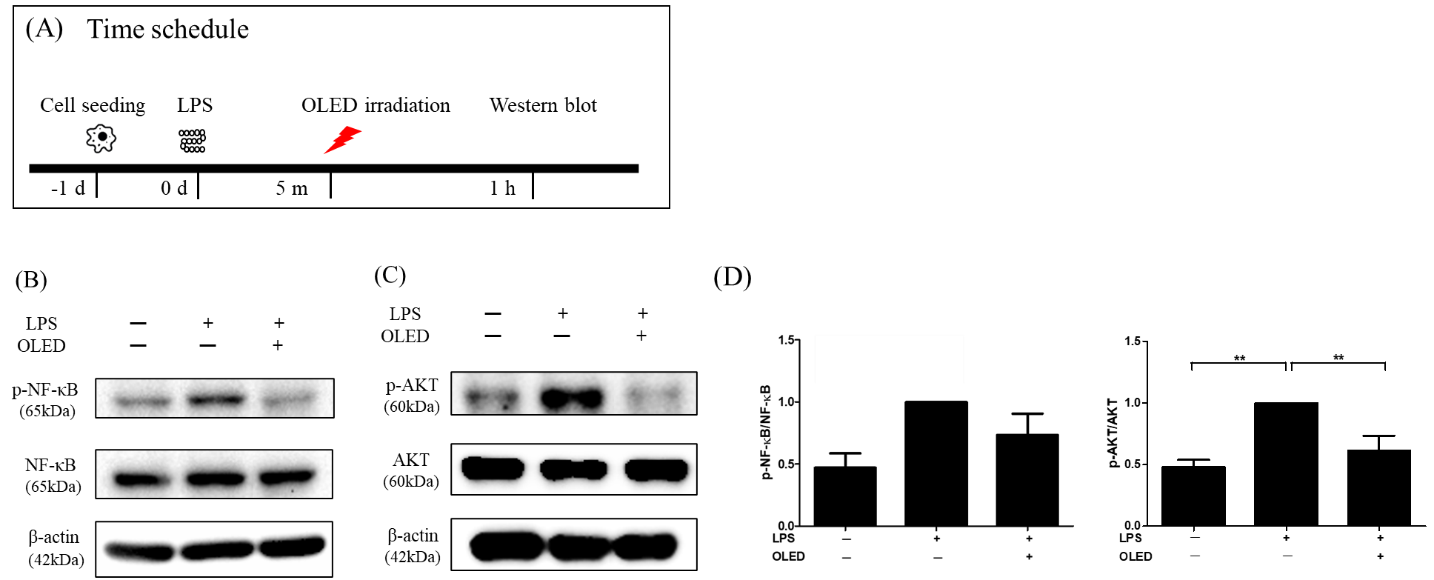
**

**Figure S3.** Western blots showing the protein expression levels of nuclear factor-κB (NF-κB), phospho-NF-κB, protein kinase B (AKT) and phospho-AKT and in lipopolysaccharide (LPS)-induced RAW 264.7 cells after organic light-emitting diode (OLED) irradiation.

**Supplementary figure 4. Uncropped images of western blot analysis (NF-κB and AKT)**

**Supplementary video 1. A video showing the flexibility and lightness of OLED patch**
